# The Efficacy of Lingual Laser Frenectomy in Pediatric OSAS: A Randomized Double-Blinded and Controlled Clinical Study

**DOI:** 10.3390/ijerph18116112

**Published:** 2021-06-06

**Authors:** Miriam Fioravanti, Francesca Zara, Iole Vozza, Antonella Polimeni, Gian Luca Sfasciotti

**Affiliations:** Department of Oral and Maxillo-Facial Sciences, Sapienza University of Rome, 00161 Rome, Italy; zarafrancesca94@gmail.com (F.Z.); iole.vozza@uniroma1.it (I.V.); antonella.polimeni@uniroma1.it (A.P.); gianluca.sfasciotti@uniroma1.it (G.L.S.)

**Keywords:** OSAS, lingual frenectomy, laser, pediatric OSAS, ankyloglossia, frenulectomy, tongue-tie, lingual frenulum, diode laser, laser therapy, oral surgery, paediatric

## Abstract

This randomized, double-blind and controlled clinical trial investigates how a diode laser lingual frenectomy can improve obstructive sleep apnea syndrome (OSAS) in pediatric patients. Background: Several authors have shown that a short lingual frenulum causes a reduction in incoming air flow and the relationship between OSAS and a short lingual frenulum. Methods: Thirty-two pediatric patients were equally randomly divided into a Study Group (SG) and a Control Group (CG). On each SG patient a polysomnography 1 (PSG1) and a lingual frenectomy were performed using a diode laser via Doctor Smile Wiser technology, power 7 W. After three months, a new polysomnography (PSG2) was performed to evaluate the lingual frenectomy efficacy in pediatric patients. The pain was assessed by a numerical rating scale (NRS) before and after surgery. The CG followed the same protocol without a lingual frenectomy but myofunctional and speech therapy were conducted to qualitatively and quantitatively improve the lingual functionality. In the SG, eight subjects (50%) had severe OSAS and eight had moderate (50%) while in the CG, three subjects had severe OSAS (18.8%) and thirteen had moderate (81.2%). Results: In the SG, 93.8% were classified as mild OSAS and 6.2% as moderate. In contrast, in the CG, 18.75% were classified as mild OSAS, 62.5% as moderate and 18.75% as severe. Conclusion: The study demonstrates how a lingual laser frenectomy can improve OSAS in pediatric patients.

## 1. Introduction

OSAS (obstructive sleep apnea syndrome) is sleep-disordered breathing (SDB) characterized by episodes of a complete or partial obstruction of the upper airways that disturbs the ventilation and sleep architecture. It is caused by various etiological factors linked to anatomical functional alterations that produce or contribute to the narrowing of the upper airways [1].

The diagnosis of pediatric OSAS should be based on the medical history of patients, which detects sleep interruptions and an upper airway collapse. The diagnosis is usually confirmed by a nocturnal polysomnography, which is the gold standard diagnostic test, followed by a clinical examination of the head and neck region [2].

The diagnosis of OSAS is based on several symptoms and can be classified according to three degrees of severity: mild, moderate and severe. The grade is assigned in relation to the apnea-hypopnea index (AHI) corresponding with the number of apnea and/or hypopnea episodes per hour of sleep. OSAS is diagnosed when the AHI is five or higher [1].

Pediatric OSAS has a prevalence between 2% and 3.5% with two peak periods [3]. The first peak is between two and eight years of age, which is due to the presence of an increase in the volume of the tonsils and adenoids. A second peak is recorded during adolescence in relation to weight gain.

The clinical examinations of many patients do not show only SDB but also a more complex symptomatology including tonsillar hypertrophy, obesity, poor body growth, adenoid facies, a contracted hard palate, micrognathy/retrognathy, anatomical and functional alterations of the lingual frenulum and arterial hypertension. OSAS also implicates a poor school performance caused by atypical daytime sleepiness, behavioral problems related to irritability or lethargy and behavioral abnormalities such as hyperactivity or aggression [4]. OSAS might determine growth failure, nocturnal enuresis, morning headaches and an increased risk of ear infections and cardiovascular diseases such as cor pulmonale [5].

Despite all of these, the etiology of OSAS remains controversial. It is now considered a multifactorial disease caused by various factors such as adenotonsillar hypertrophy, allergic rhinitis, obesity, craniofacial anomalies, genetic and/or congenital neuromuscular diseases, cleft palate, a contracted upper jaw or changes in lingual posture [4,6,7].

Removing the risk factors and an early detection of clinical aspects may facilitate the diagnosis leading to a great treatment plan that can quickly improve the clinical condition of the patients. The pediatric dentist could make an early diagnosis during the clinical examination by evaluating the presence of a short lingual frenulum and an oral malocclusion [8].

Due to the wide variety of clinical manifestations related to OSAS, a multidisciplinary approach is the best way to treat this disorder and involves different practitioners such as pediatricians, otolaryngologists, maxillofacial surgeons and pediatric dentists. The treatment plan also includes several therapeutic methods such as an adenotonsillectomy, CPAP (continuous positive airway pressure), a correct management of body weight, orthodontic treatment and the correction of ankyloglossia conditions [8,9,10].

The main goal of therapy is to eliminate the risk factors of an upper airway collapse thus reducing the risk of persisting OSAS.

A functional limitation of the tongue might have a few consequences both in infants and in teenagers. Functional alterations lead to different swallowing and chewing patterns, causing an incorrect coordination of the muscles and an anterior position of the tongue with possible malocclusions [9,10,11,12]. Therefore, several professional pediatricians, oral surgeons, orthodontists and speech therapists can be required for the treatment of this condition, which has a prevalence rate of 4% to 10% among newborns with a male to female ratio of 2:1 [9,10,11,12]; ethnicity is not a risk factor [13,14].

The diagnosis of a pathological lingual frenulum is based on quantitative or qualitative classification criteria. Kotlow and Ruffoli provided the quantitative classification; it is widely used in clinical practice. Instead, the qualitative classification describes the function of the tongue (tool for lingual frenulum function (ATLFF)) [15]. Moreover, the most recent Bristol Tongue Assessment Tool (BTAT) [16,17] evaluates four anatomical functional characteristics and is easier to use.

Quantitative assessments are represented by a measurement of Kotlow’s free tongue (normal ≥ 16 mm) [18], the opening of the mouth with the tip of the tongue on the incisal papilla (MOTTIP, normal ≥ 23 mm) [19] and the maximum opening of the interincisal mouth (normal MY ≥ 35 mm) [20].

Surgical procedures are the conventional methods for managing a pathological lingual frenulum from a scalpel, a more traditional technique, to electrosurgery or laser surgery. The latter is the most widespread technique nowadays.

According to Vozza et al., laser surgery offers several advantages such as disinfection, precise incisions, minimal damage to adjacent tissues, a hemostatic effect, improved postoperative progression, no need for sutures and a reduction in the dose of local anesthetic; a goal to be achieved especially in pediatric patients [21,22,23,24,25].

In the literature, there are several studies demonstrating the benefits of the use of a laser but most of them are not sufficiently randomized or do not evaluate the postoperative quantitative improvement of the frenulum [20].

The aim of this study is to evaluate the efficacy of a lingual frenectomy performed through diode lasers to improve the length of frenulum and the severity of OSAS in pediatric patients.

## 2. Materials and Methods

### 2.1. Trial Design, Research Strategy and Inclusion Criteria

An open, randomized, double-blinded, controlled clinical study was conducted at the Policlinico Umberto I, “Sapienza” University, Hospital of Rome, Head and Neck Department, Pediatric Dentistry Unit to evaluate the efficacy of a lingual frenectomy through diode laser technology in pediatric patients with OSAS and comparing them with a group of control cases from August 2019 to September 2020.

This study used a randomized controlled experimental design based on the guidelines recommended by the Consolidated Standard of Reporting Trials (CONSORT 2010).

The Institutional Review Board of territorial NHS facilities (n. 250620) approved the study. The protocol of this study was drawn up in accordance with the Standards of Good Clinical Practice of the European Union in accordance with the 1975 Declaration of Helsinki.

### 2.2. Participants

The enrollment of patients was performed following specific inclusion criteria: a diagnosis of OSAS and a short lingual frenum, a chronological age (range from 4 to 13 years), the presence of a pathological lingual frenulum (class III–IV–V by Kotlow; Grades 2 and 3 by Ruffoli), no previous frenectomy or frenulotomy, no systemic pathology and signed informed consent. The exclusion criteria were: absolute or relative contraindications to the administration of a local anesthetic, a non-pathological lingual frenulum (Kotlow class I–II; Ruffoli grade 1), a previous frenectomy or frenulotomy and patients whose legal guardians did not give informed consent to join the study. Fifty patients with OSAS and a short lingual frenulum were assessed for eligibility. Eighteen were excluded (not matching the inclusion criteria (*n* = 12), refusing to participate (*n* = 3) or for other reasons (*n* = 3)).

A total of 32 female and male pediatric patients were then finally enrolled in the study.

Among the 32 patients, 14 patients were female (43.8%), 18 were male (56.3%) and they were aged between 4 and 13 years. 

The disorders were diagnosed through clinical and radiologic examinations and with the reference intervals of the Kotlow classifications (distance of the tip of the tongue–lingual insertion of the frenulum) and of Ruffoli (total length of the frenulum). In addition, a functional evaluation was performed (lingual movements of protrusion raising towards the palate) with the use of the device Quick Tongue Tie Assessment Tool^®^, Oralfacial Myology [17].

The Kotlow and Ruffoli classification systems are based on the quantitative evaluation of the lingual frenulum. The qualitative classification systems, on the other hand, make it possible to analyze the lingual functionality.

A polysomnography was requested to complete the diagnosis of OSAS.

The legal guardians of the participants in the study signed an informed consent document to participate in the study [26]. 

### 2.3. Sample Size and Randomization: Sequence Generation

Thirty-two male and female OSAS patients aged between 4 and 13 (M = 7.00, SD = 2.49) were randomly subdivided into two groups, a study group (SG) and a control group (CG) (Table 1 and Table 2). The randomization was done according to a computer-generated series (Research Randomizer^®^ free resource).

The SG consisted of 16 patients (7 were female and 9 were male; M = 6.19; SD = 2.40), with corresponding percentages of 43.75% female and 56.25% male. In the SG, eight subjects (50%) had severe OSAS and eight had moderate OSAS (50%).

The SG (a prelingual and postlingual frenectomy polysomnography conducted through laser technology) patients (*n* = 16) were examined through a clinical and radiographic examination together with an otolaryngological evaluation.

This group of patients underwent a visit conducted by an otolaryngologist specialist together with a polysomnography 1 (PSG1). Subsequently, the SG received a lingual frenectomy through Doctor Smile Wiser technology provided by Doctor Smile^®^ (Lambda Spa, Vicenza, Italy). Local anesthesia and laser cutting of the lingual frenulum were performed by the oral surgeon, stabilizing the tongue using hemostatic forceps without applying sutures. This medical device (Medical class/Laser II B/4) emits a laser beam with a wavelength of 980 nm; each application was performed at a peak power of 7.5 W in a continuous mode with a frequency up to 25 Hz (Table 3).

A surgeon, skilled in laser therapy, performed the laser lingual frenectomy surgery at the Policlinico Umberto I, “Sapienza”, University Hospital of Rome, Head and Neck Department, Pediatric Dentistry Unit. The practitioner also instructed patients on myofunctional exercises to perform at home.

After three months, a new polysomnography (PSG2) was conducted to evaluate the effectiveness of the lingual frenectomy in pediatric patients with OSAS.

The CG consisted of 16 patients (7 were female and 9 were male; M = 7.81; SD = 2.37) with corresponding percentages of 43.75% female and 56.25% male. In the CG, three subjects had severe OSAS (18.8%) and 13 had moderate OSAS (81.2%).

The CG (premyofunctional and postmyofunctional therapy polysomnography) protocol provided for a dental and orthodontic clinical evaluation through an objective and radiographic clinical examination and an otolaryngological evaluation.

This group of patients was visited by an otolaryngologist specialist and was submitted to the first polysomnography 1 (PSG1). Subsequently, myofunctional and speech therapy was conducted to improve the lingual functional capacities qualitatively and quantitatively. After three months, a new polysomnography (PSG2) was performed to evaluate the effectiveness of myofunctional and lingual speech therapy in pediatric patients with OSAS.

### 2.4. Blinding

In accordance with the design of the study in both groups, the SG and the CG, neither the health worker assigned to the intervention conducted nor the patient who underwent the treatment became aware of the double-blind assignment of the group.

### 2.5. Study Setting and Interventions

The same surgeon, skilled in laser therapy, always performed each intervention.

**Figure 1:**
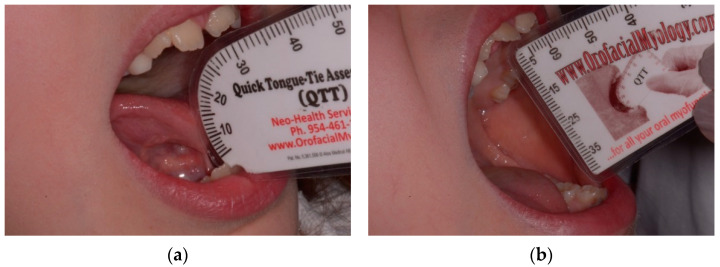
Quantitative variables at T0: (**a**) Kotlow; (**b**) MAB.

**Figure 2:**
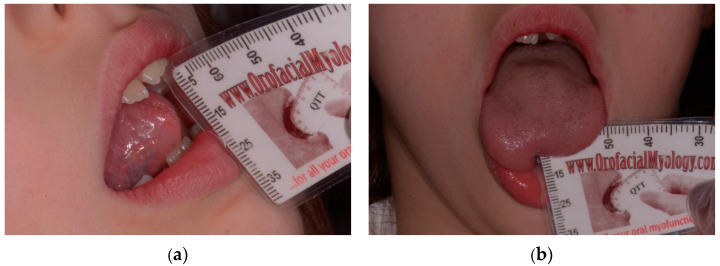
Quantitative variables at T0: (**a**) MOTTIP; (**b**) Protrusion.

All patients were placed in a follow-up program for a functional and instrumental evaluation at T0 and T1 (28 days), during which the following parameters were recorded: Kotlow, maximum opening of the mouth (MAB) (Figure 1), distance between the interincisal margins with the tip of the tongue positioned at the level of the superior retroincisive papilla (MOTTIP) and lingual protrusion (Figure 2). Furthermore, a qualitative functional assessment was performed with specific scales such as the Assessment Tool for Lingual Frenulum Function, Bristol Tongue Assessment Tool and the degrees of lingual function.

The quantitative parameters were assessed using the Quick Tongue Tie Assessment Tool^®^, Oralfacial Myology [17]:

This single use tool was used to evaluate the variables above.

Data were carried out by a different health professional in order not to alter the final result of the double-blind study. Exercises were explained by the surgeon to patients through the support of a representative scheme then each patient exercised with them at home following the specific protocol [23].

A pain evaluation was registered by the same blinded examiner immediately before (T0) and at the end of the treatments (T1). The SG patients were also given a clinical diary in order to estimate the painful symptoms in the days following the surgery at 24 h, 48 h, 72 h, 14 days and 28 days after surgery.

The numerical rating scale (NRS) was adopted for the pain assessment. The NRS pain evaluation system was successfully adopted due to its good reliability and accuracy, as demonstrated in many clinical studies [27,28].

The examiner requested that the patient indicated the level of pain sensation on a scale from 0 to 10 where 2 indicated discomfort and 10 was the maximum pain felt.

After the treatment, all of the patients received conventional therapy for the resolution of the short lingual frenulum.

### 2.6. Statistical Analysis and Data Analysis

The data analysis was carried out with the SPSS version 24 statistical processing program. An analysis of variance for repeated measures was carried out in order to evaluate the different experimental parameters of the values of the subjects (Kotlow, MAB, MOTTIP and Protrusion).

An analysis of variance between the subjects was conducted in order to find the difference between the various experimental conditions.

The characteristics of the patients of the two groups were measured at the baseline (sex, age, intensity of the disease) and they were compared using the chi-squared test and the Mann–Whitney test for metric variables in order to avoid any bias in the formation of the groups.

The chi-squared test was used to evaluate the association between the intensity of the disease (mild, moderate and severe) and the type of followed therapy (laser or myofunctional). The Mann–Whitney test was used to compare the mean scores of the patients in the experimental and control groups in relation to the quantitative variables (Kotlow, MAB, MOTTIP, Protrusion). Finally, the Wilcoxon test was conducted to establish for each group whether the values of Kotlow, MAB, MOTTIP and Protrusion reported significant variations between T0 and T1.

## 3. Results

An improvement from T0 to T1, was found in the parameters mentioned above.

The comparison between PSG1 and PSG2 indicated that there was an improvement in the parameters assessed with the polysomnographic examination.

The pain recording, an estimated value for the SG, showed no pain as early as 72 h after surgery.

### 3.1. Participants

A total of 32 patients took part in the study (14 were female (43.8%); 18 were male (56.3%), aged between 4 and 13 years (M = 7.00; SD = 2.49). Among these, 16 were randomly assigned to the experimental group (7 females and 9 males; M = 6.19; SD = 2.40) and 16 to the control group (7 females and 9 males; M = 7.81; SD = 2.37). In the study group, eight subjects (50%) had severe OSAS and eight had moderate OSAS (50%), while in the control group three subjects had severe OSAS (18.8%) and thirteen had moderate OSAS (81.2%).

The results were considered significant if they showed a *p*-value < 0.05.

### 3.2. Comparison between Groups at the Baseline

There were no significant differences between the groups in relation to the distribution by gender (Fisher’s exact test χ2 = 0; *p* = 1.000), age (U = 93.5; *p* = 0.212) and disease intensity (Fisher’s exact test χ2 = 3.463; *p* = 0.135) at T0.

Furthermore, during the surveys carried out at T0, the groups did not show significant differences in the scores of the quantitative variables included in the study: Kotlow (U = 99.5; *p* = 0.270), MAB (U = 106.5; *p* = 0.407), MOTTIP (U = 116; *p* = 0.649) and Protrusion (U = 119.5; *p* = 0.747). (Table 4).

### 3.3. Comparison between Groups in the Intensity of OSAS Pathology Following the Intervention

A comparison was made between the cases and controls in relation to the intensity (mild, moderate or severe) of the OSAS pathology. The results showed a significant association between the therapy and the intensity of the disease (χ^2^ = 18.364; *p* < 0.0001). The analysis of the standardized residues established why the SG reported a more frequent mild intensity of the disease than the CG, which instead reported a more moderate or severe intensity of OSAS.

Specifically, in the group of children who underwent laser therapy (SG), 93.8% were classified as mild OSAS and 6.2% as moderate OSAS (Table 5).

In contrast, in the group of children who underwent myofunctional therapy (CG), 18.75% were classified as mild OSAS, 62.5% as moderate OSAS and 18.75% as severe OSAS (Table 6).

### 3.4. Comparison between Groups With Respect to Quantitative Variables

The Mann–Whitney test showed no significant differences in the scores of the quantitative variables included in the study and measured at T1: Kotlow (U = 96.5; *p* = 0.233), MAB (U = 122; *p* = 0.820), MOTTIP (U = 115.5; *p* = 0.637) and Protrusion (U = 85; *p* = 0.103). (Table 7).

The results relating to the study group (SG) showed that between T0 and T1 there was a significant increase in the average scores of all of the considered variables: Kotlow (Z = −3.521; *p* < 0.001), MAB (Z = −3.436; *p* < 0.01), MOTTIP (Z = −3.536; *p* < 0.001) and Protrusion (Z = −3.527; *p* < 0.001). (Table 8).

Similarly, in the control group there was also a significant increase in all of the scores relating to the considered variables: Kotlow (Z = −3.531; *p* < 0.001), MAB (Z = −3.088; *p* < 0.01), MOTTIP (Z = −3.412; *p* < 0.01) and Protrusion (Z = −3.426; *p* < 0.01). (Table 9).

## 4. Discussion

Several studies assumed the existence of a relationship between OSAS and a short lingual frenulum but the real innovation that characterized this study was the results, which demonstrated a clear correlation between a short lingual frenulum and OSAS. Surgical treatment of the condition of ankyloglossia with the use of diode laser technology in pediatric patients led to a complete or partial resolution of OSAS.

In this study, only patients with a pathological lingual frenulum (Kotlow class III–IV–V; Ruffoli grade 2 and 3) and with a certain diagnosis of OSAS were enrolled in order to obtain a greater accuracy of the efficacy of this protocol.

A pathological frenulum causes a reduction in lingual mobility, which therefore cannot perform its functions correctly [29,30,31].

The altered lingual posture in fact determines a different direction of growth of the jaw bones, which, together with other predisposing factors, could lead to orofacial dysmorphosis with a reduction in the influx of incoming air with consequent respiratory problems, orthodontic problems and altered habits such as oral breathing, aerophagia and the forward positioning of the tongue [29,30]. Therefore, it decreases the size of the upper airway support and progressively increases the risk of an upper airway collapse during sleep [30,31].

As demonstrated by Lee et al., changes in orofacial growth related to factors including a short lingual frenulum lead to SDB and mouth breathing very early in life [30].

The habit of oral breathing induced by ankyloglossia could be at the basis of the relationship between the presence of the pathological lingual frenulum and the onset of OSAS [32].

In a recent report, Guilleminault et al. retrospectively studied 150 children with OSAS, 63 of them with a short frenulum. The conclusions showed that an untreated short frenulum at birth is associated with OSAS in old age and hence systematic screening for OSAS should evaluate this anatomical anomaly [32].

In the scientific literature, numerous studies have analyzed the relationship between the presence of a short lingual frenulum and a dentoalveolar malocclusion [33].

The presence of a short lingual frenulum, in fact, limits the possibility of a lingual upward movement. This consequently leads to the onset of abnormal swallowing and an anterior open bite, which perpetuates the incorrect swallowing pattern with a continuous forward thrust of the tongue with a modification of the dental occlusion. This defect can be solved with an orthodontic approach.

The recognition of the negative impact of a short lingual frenulum has led specialists to develop sophisticated protocols for the study of infants and children with a short lingual frenulum [34,35].

Zaghi et al. demonstrated that a lingual frenuloplasty with myofunctional therapy is safe and potentially effective for the treatment of mouth breathing, snoring, clenching and myofascial tension in appropriately selected patient candidates [36].

The pathological lingual frenulum, in the most complex cases, can lead to problems that can be solved only and exclusively with the collaboration of various professional figures including a pediatric dentist, orthodontist, otolaryngologist and speech therapist. The last two figures should intervene alongside the dentist in the medical team in case of prolonged respiratory problems such as OSAS or in case of bad habits or dyslalias.

For this reason, the treatment of the condition of ankyloglossia can aid in the improvement and in a few cases in the resolution of OSAS.

Although many authors have conducted clinical studies demonstrating the correlation between OSAS and a short lingual frenulum, this randomized, double-blind, controlled clinical trial was the first to evaluate the efficacy of a lingual frenectomy in the partial or complete resolution of respiratory disease.

The dentist plays a key role in the diagnosis and treatment of OSAS in children. The role of the pediatric dentist and orthodontist, in fact, in the therapeutic approach to pediatric OSAS is to act as a “sentinel” able to detect the first signs of the disease and note down elements regarding school performance, ability of concentration, the possible presence of nocturnal enuresis, inappetence, obesity and recurrent infections of the airways and OSAS. He can intervene therapeutically, evaluating the cranium-facial area, breathing, elongated face, small and retracted chin, dental crowding, high palate and narrow, lingual posture and the length and mobility of the lingual frenulum for an immediate referral to the otolaryngologist and to actively participate in therapy [37].

OSAS therapy should be multidisciplinary including AT, continuous positive pressure mechanical ventilation (CPAP), weight loss in overweight children and mandibular advancement devices (MAD) [38]. These alternatives are poorly tolerated in children and are rarely considered as a primary therapy.

Recommendations published by the National Institute of Health’s National Guidelines System (SNLG) in a 2008 publication state that orthodontic intervention should be considered a therapeutic option before or at the same time as continuous positive airway pressure (CPAP) treatment [39].

The *National Guidelines For The Promotion Of Oral Health And The Prevention Of Oral Diseases In Developmental Age*, Ministry of Health, 2008 edition, indicate with a high level of evidence and force that “Children with predominantly oral breathing and contracted upper jaws benefit from orthopedic expansion of the maxilla” [40].

Rapid maxillary expansion (RME) has a positive effect on the upper airways of the pharynx, is capable of modifying the nasal-maxillary soft tissue complex, decreases nasal resistance and allows for better oxygen saturation [41,42,43].

Mandibular advancement guarantees the expansion of the retrolingual space and therefore the advancement of the position of the tongue.

It is necessary to emphasize the importance of myofunctional therapy associated with orthodontic therapy to ensure the stabilization of the effects of orthodontic treatment and to restore the correct nasal respiratory process.

In a few cases, however, orthopedic therapy of the upper jaw alone is not decisive for the treatment of OSAS when it is caused by neuromuscular changes such as a low tongue posture due to ankyloglossia.

The treatment of ankyloglossia consists of a frenectomy, which can be performed by various methods including the traditional surgical method (cold blade), electrocautery and laser technology, which respects the principles of minimally invasive dentistry and is among the most innovative of the therapies [44].

The laser offers numerous benefits including proper hemostasis, reduced operating times, easier access, disinfection of the surgical field, precision of the incision, minimal damage to the surrounding tissues, improved tissue healing, reduced inflammation, pain control and a more comfortable postoperative course and complete patient acceptance, especially in the pediatric patient [22,45].

Reddy et al. reported five clinical cases of a short lingual frenulum; three of them were treated with electrocautery therapy, one with a diode laser and one with a traditional surgical technique (scalpel 15c). After a follow-up of 7 and 30 days, better tissue management was observed in the laser-treated cases compared with the traditional surgical techniques, which caused more pain and swelling. The use of laser for the treatment of a frenectomy can be considered safe and reliable with pain reduction and an improvement of tongue function [46].

Derikvand et al. demonstrated several advantages of laser technology such as improved healing and reduced postoperative complications [47].

Barot et al. reported very positive results after a frenectomy with laser technology (wavelength: 810 nm; fiber diameter: 200 µm used in direct contact with the tissue; power 2 W in a continuous mode and a focused beam). Through this protocol, the authors achieved complete healing, an increase of >16 mm in tongue mobility and speech improvement after therapy [48].

Sfasciotti et al. demonstrated the efficacy of a lingual frenectomy with diode laser technology through a qualitative and quantitative evaluation in pediatric patients [28]. In this study, one hundred and twenty-five pediatric patients were recruited: 100 with a lingual pathological frenulum randomly divided into four operating groups; the other 25 with a borderline pathological frenulum were recruited as a control group. Each patient was included in a follow-up program for a quantitative and qualitative evaluation. The authors reported an increase of the quantitative parameters of circa 10 mm and a reacquired full functionality of the tongue.

The lack of traditional surgical instruments and the hemostatic effect of the laser resulted in bloodless surgery and in the lack of need for affixing sutures, which are annoying in the postoperative course especially when performing myofunctional exercises.

These advantages are all very useful in pediatric patients who live the experience of surgery in a more anxious way than adults do. Children, in fact, gladly accept lasers; the vision of the light beam in the preparatory phase for the intervention generates curiosity and can help in increasing patient compliance in the intraoperative phase [49].

The postoperative course should always be accompanied by the execution of myofunctional exercises in the home environment.

### Limitations of the Study

The evaluation of pain is extremely subjective.

Inconsistent levels of compliance by the patients could have influenced the improvements of the parameters unevenly.

In accordance with this study, further randomized studies with greater samples are essential to understand the efficacy of a lingual frenectomy in pediatric OSAS.

## 5. Conclusions

The results obtained from this study indicated how the condition of the short lingual frenulum affected the severity and intensity of the OSAS pathology. This trial demonstrated that diode laser lingual frenectomy therapy can improve the severity of OSAS in pediatric patients.

Therefore, an early diagnosis and treatment of the condition of ankyloglossia associated with myofunctional therapy should be indicated in pediatric subjects with sleep apnea problems.

From the results obtained in this study, the pediatric dentist could be considered a sentinel in the early diagnosis of OSAS during a historical and physical examination by investigating not only malocclusions associated with OSAS but also by recording qualitative and quantitative measures of the lingual function.

## Figures and Tables

**Table 1:** Summary of data for each group.

Group	Number of Patients	Laser Surgery	Myofunctional and Speech Therapy
Study Group (SG)	16	Yes	Yes
Control Group (CG)	16	No	Yes

**Table 2:** Sociodemographic table.

Sociodemographic Index	Patients	Study Group	Control Group
Nationality	Italian	14	13
	Other	2	3
Gender	Male	7	9
	Female	9	7
Age	4–5	2	3
	6–8	4	7
	9–10	9	4
	11–13	1	2
Instruction	Kindergarten	2	3
	Elementary	10	11
	Middle	4	2

**Table 3:** Setting of the device.

Name	Doctor Smile^®^ (Wiser Laser Evolution)
Wavelength	980 nm
Highest output power	7.5 pulsed watts
Modality of pulse	Variables pulse wave
Frequency	Up to 25 Hz
Medical class/laser	B/4
Tissue optimize pulsing	Adjustable power for every type of soft tissue
Diameter fiber	300 µm
Duration of intervention	300 s
Number of sessions	Single surgery session
Production site	Italy-Brendola (VI)

**Table 4:** Comparison between the SG and the CG at T0 in relation to the quantitative variables.

Quantitative Variables	SG T0	CG T0	Mean Score	SD
Kotlow	17	17.63	17.315	0.445477
MAB	38.13	39.31	38.72	0.834386
MOTTIP	20.38	19	19.69	0.975807
Protrusion	18.56	17.5	18.03	0.749533

**Table 5:** Intensity of OSAS of the SG at T1.

Intensity of OSAS	OSAS SG T1
Mild	93.8
Moderate	6.2
Severe	0

**Table 6:** Intensity of OSAS of the CG at T1.

Intensity of OSAS	OSAS CG T1
Mild	18.75
Moderate	62.5
Severe	18.75

**Table 7:** Comparison between the SG and the CG at T1 in relation to the quantitative variables.

Quantitative Variables	SG	CG	Mean Score	SD
Kotlow	24.56	22.31	23.435	1.59099
MAB	42.12	41.63	41.875	0.346482
MOTTIP	25.44	24.13	24.785	0.92631
Protrusion	25.12	21.81	23.465	2.340523

**Table 8:** Comparison of T0 and T1 of the SG in relation to the quantitative variables.

Quantitative Variables	T0	T1	Mean Score	SD
Kotlow	17	24.56	20.78	5.345727
MAB	38.13	42.12	40.125	2.821356
MOTTIP	20.38	25.44	22.91	3.57796
Protrusion	18.56	25.12	21.84	4.63862

**Table 9:** Comparison of T0 and T1 of the CG in relation to the quantitative variables.

Quantitative Variables	T0	T1	Mean Score	SD
Kotlow	17.63	22.31	19.97	3.30926
MAB	39.31	41.63	40.47	1.640488
MOTTIP	19	24.13	21.565	3.627458
Protrusion	17.5	21.81	19.655	3.04763

## Data Availability

Data available upon request due to restrictions. The data presented in this study are available upon request. The data is not publicly available for privacy reasons.
